# Synthesis, electrospinning, and molecular docking of poly(methyl methacrylate) Schiff bases and their applications as polymeric antimicrobial agents and for dye removal

**DOI:** 10.1007/s11356-023-30043-4

**Published:** 2023-09-28

**Authors:** El-Refaie Kenawy, Ahmed R. Ghazy, Ahmed F. Al-Hossainy, Mohamed Bishr, Mohamed M. Azzam

**Affiliations:** 1 Polymer Research Group, Chemistry Department, Faculty of Science, Tanta University, Tanta, 31527 Egypt; 2 Laser Laboratory, Physics Department, Faculty of Science, Tanta University, Tanta, 31527 Egypt; 3 Chemistry Department, Faculty of Science, New Valley University, El-Kharga 72511, New Valley, Egypt

**Keywords:** Poly(methyl methacrylate) nanofibers, Dye removal, Antimicrobial polymers

## Abstract

The antibacterial activity of a variety of modified poly(methyl methacrylate) Schiff bases against common microbial infections and removal of methylene blue (MB) dye were screened. The Schiff bases were synthesized from the reaction of the modified (PMMA) with vanillin (PMMA)^Van^ and cinnamaldehyde (PMMA)^Cin^. By using Fourier transformer infrared (FT-IR), X-ray diffraction analysis (XRD), thermogravimetric analysis (TGA), and scanning electron microscopy (SEM), the structures of the nanofibers of the synthesized Schiff bases were confirmed. The modified Kirky–Bauer method was used to screen the antibacterial activities of all the obtained materials against various bacterial species, including gram-positive bacterial (*Bacillus subtilis* (4k1p),* Staphylococcus aureus*), Gram-negative bacteria (*Escherichia coli* (7ab3),* Pseudomonas aeruginosa*). Inhibition zones against gram-positive bacteria ranged in diameter from 7 to 14 mm, whereas for the Gram-negative bacteria, the inhibition zones found to be ranged between 6 and 13 mm. With a minimum bactericidal concentration (MBC) of 8 mg/mL and a minimum inhibitory concentration (MIC) of 2 mg/mL, (PMMA)^Van^ shown the greatest antibacterial activity. Lastly, molecular docking research was done to better understand the interactions between this series' targets and inhibitors for (PMMA)^Van^ and (PMMA)^Cin^ (4k1p and 7ab3). Molecular modeling of these surface-adsorbed polymers indicated that (PMMA)^Van^ binds more strongly with Nitrogen than does (PMMA)^Cin^ through extra hydrogen-bonding interactions. All the developed materials were evaluated for the removal of 0.1 g/L methylene blue dye (MB) from an aqueous solution. The elimination percentage of MB dye ranged from 26.67% by using 0.05 g powder of (PMMA)^Cin^ to 85.63% by employing 0.05 g nanofibers of (PMMA)^Van^.

**Graphical Abstract:**

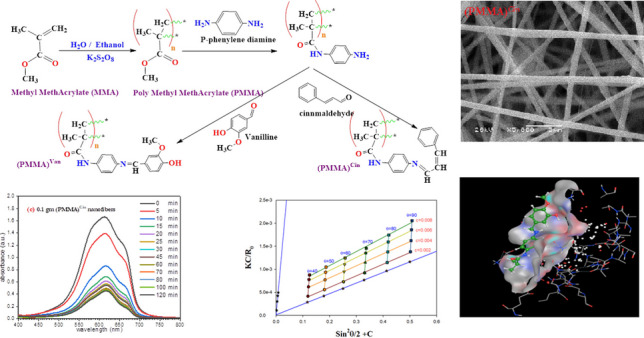

## Introduction

Drinking pathogenic microorganisms contaminated water is the reason for serious diseases such diarrhea, cholera, and typhoid (Fawell and Nieuwenhuijsen 2003). It has become vital to create safe, affordable, and simply applied technology to remove harmful bacteria from drinking water (El-Aassar et al. 2019a; El-Kowrany et al. 2016). Unique materials with useful features have been created by composite research (Berber et al. 2010; El-Aassar et al. 2019b). A number of processes are used to remove wastewater dyes, including adsorption, advanced oxidation, coagulation, and membrane separation (Gupta 2009). Biological, chemical, and physical elimination methods can all be grouped together (Ghoreishi and Haghighi 2003). Industries use adsorption as one of the most efficient ways for wastewater treatment to remove dangerous organic and inorganic contaminants (Kant 2012; Kenawy et al. 2019a). Schiff bases’ chemical structure, which is crucial in the management of microorganisms, has demonstrated a broad biological function. Moreover, because of their powerful adsorption effects and durability, Schiff bases were utilized in a variety of manufacturing applications for dye removal (Zhao et al. 2020). Biological effects of Schiff bases range widely (Kenawy et al. 2019b). Having qualities that are antibacterial, antifungal, antimalarial, antiviral, antipyretic, and anti-inflammatory (Azaam et al. 2018; Da Silva et al. 2011). A quick, affordable, and adaptable method for creating nanofibers from polymeric solutions is electrospinning. Although this method was developed in 1934, it has not been thoroughly investigated until the past 10 years (Naebe et al. 2010). Even though the bonding of surface hydrogen may be the main operator for the changes in the adsorbed polymers properties, interfacial interactions might be challenging to measure. In order to better understand the role that these interactions can play, poly(methyl methacrylate) (PMMA), poly(vinyl acetate) (PVAc), and poly(methyl methacrylate), two chemically equivalent polymeric systems with identical chemical formulas but different functional groups, were examined (Mortazavian et al. 2016). Various functional groups lead to adsorption behaviors that are a little bit different. Although thermal behavior studies of the adsorbed polymeric systems have shown that both polymers have the capacity to absorb surface hydrogen bonds, they also indicate that the thermal properties changes following adsorption are distinct and statistically significant (Khatiwada et al. 2013). Compared to recent discoveries on PVAc, earlier research on PMMA demonstrates noticeably larger changes in the glass transition after adsorption. It is unclear how or whether hydrogen bonding alone might explain the differences in thermal characteristics given the similarity of the chemical structures (Chan and Chu 2001).

In this study, the relative ketones compounds of the polymers are compared to their bulk counterparts, and the synthesis and characterization characteristics of bulk PMMA Schiff base are examined. This study focuses on the structural characterization of the tightly bound region of the adsorbed polymers and direct comparisons of these adsorbed polymers at similar absorbed amounts, molecular weights, and environmental conditions. The interaction of the polymers with other ketone compounds was also studied using molecular simulations to spot modifications in the microscopic surface contacts that affect the structural characteristics. The results show that whereas both (PMMA)^Van^ and (PMMA)^Cin^ may hydrogen-bond with nitrogen atoms and share structural similarities, (PMMA)^Cin^ exhibits stronger hydrogen-bonding interactions as a result of its side-chain groups orientation.

## Experimental

### Materials and instruments

The compounds that have been employed are mentioned in Table 1 and have not been further purified. FT-IR spectra were measured using KBr pellets and a Perkin-Elmer 1430. SEM photos were taken using a JEOL JSM-IT100 running at 20 kV. An X-ray diffraction pattern at a 2θ range of 5–80 was obtained using a Rigaku X-ray diffractometer with Cu K (*d* = 1.540°A). The second virial coefficient, radius of gyration and molecular weight were determined using static light scattering experiment. Germany’s IKA Corporation supplied the rotary evaporator. Germany’s binder supplied the hoover oven. Model E03-001 of the electrospinner, produced in China by Qingzi Nano, scanned under Biochrome Libra S50PC control. Absorption spectra were measured using a UV/Vis spectrophotometer with a 190–1100 nm wavelength range. Thermogravimetric analysis (TGA) analysis was carried out using a Perkin Elmer TGA 4000 thermogravimetric analyzer. Samples of 5–10 mg were placed into alumina crucibles and scanned (from 50 to 800 °C) at a rate of 30 °C/min while being surrounded by nitrogen atmosphere at a flow rate of 20 mL/min.

**Table 1:** List of the used chemical

Chemical	Molecular formula	Supplier
Methyl methacrylate (MMA) monomers	C_5_H_8_O_2_	Across organics
acrylonitrile (AN) monomers	C_3_H_3_N	Across organics
Potassium persulfate	K_2_S_2_O_8_	Fisher Co (UK)
Dimethylformamide (DMF)	C_3_H_7_NO	Fisher Co (UK)
Vanillin	C_8_H_8_O_3_	El goumhouria Chem. (Egypt)
*p*-phenylene diamine	C_6_H_8_N_2_	Across organics

### Polymerization of methyl methacrylate

Precipitation polymerization was used to create (PMMA) polymers in accordance with the procedure outlined by Mohy Eldin et al. (Eldin et al. 2017). A 70/30 water/ethanol solution was created by combining distilled water and ethanol. A 10% monomers/solvent ratio of MMA was added to the produced solvent at room temperature. The mixture was gradually added amounts of potassium persulfate (K_2_S_2_O_8_) (0.05 M) to start the polymerization process. On a hotplate, the polymerization process took place at a temperature of 55 °C for 4 h. Following polymerization, the end product was separated by 10,000 rpm centrifugation. The finished item was then cleaned with a solution of ethanol and distilled water before being dried for 24 h at 60 °C.

### Synthesis of aminated PMMA

*p*-Phenylene diamine was mixed with a suspension of poly methyl methacrylate at a concentration of 0.1 g/ml. For 24 h, the reaction mixture was agitated at 90 °C. To get rid of the extra ethylene diamine, the product was filtered out, rinsed with distilled water, and then treated with ethanol. The product was vacuum-dried for 2 days at 40 °C.

### Synthesis of Schiff bases of PMMA

**Scheme 1:**
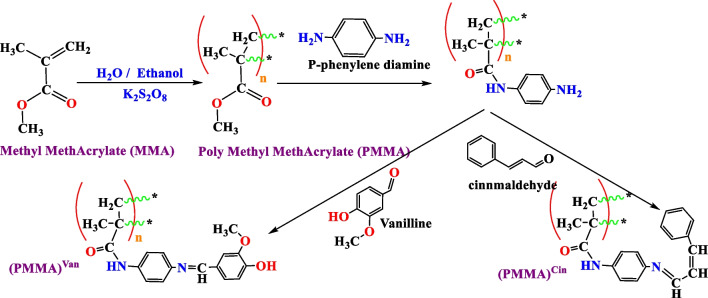
Synthesis of PMMA Schiff bases

Aminated PMMA was dissolved in DMSO in 500 mL of two-necked flasks at 120 °C. The combination was then agitated at 120 °C until a clear solution was achieved. Vanillin and cinnamaldehyde were then added, and the mixture were heated at 90 °C for 15 min (14 h). Ethyl alcohol was used to filter and wash the product as shown in Scheme 1.

### Electrospinning of modified PMMA

Six grams of polyacrylonitrile (PAN) (as a co-spinning agent) and 2 g of PMMA suspension were combined with DMF as the solvent. The settings for electrospinning were voltage 11.5 kV, electrode separation 9 cm, and temperature 20 °C. Twenty milliliters polypropylene syringes were used in the electrospinning setup (nano NC, ESR100D, Korea) used in this work to load the electrospinning solution. The electrospun nanofibers were collected on a flat metal screen that is detachable, electrically grounded, and movable to the desired height and direction. A gap of 10 to 30 cm was kept between the nozzle and the collection screen while voltages between 15 and 30 kV were produced using a high-voltage power supply. The quantity and flow rate of the polymer solution leaving the spinneret were managed by a pump. The spinneret was a blunt metal needle with an exterior diameter of 16 mm, an interior diameter of 1 mm, and a length of 50 mm. With varying the polymer content, needle tip-to-collector distance, flow rate, and finally the provided voltage, it was intended and determined for each tested solution that the electrospinning parameters be optimized to obtain nanofibers free of defects. The solution was first manually pushed until it reached the needle tip after being placed into the syringe. Every experiment was conducted at room temperature. No further coating of any kind was required to collect the electrospun mats since the correct electrospun membranes were rather simply removed from the aluminum foil used to cover the collector. The electrospinning of approximately 15 mL of polymer solution produced the necessary membranes. The latter was then using a razor blade removed from the collection and stored in a plastic bag that was hermetically sealed. On the collector, electrospun nanofibers were placed and gathered.

### Antimicrobial assay

A modified Kirky–Bauer diffusion method was used to incorporate the antibacterial properties of these polymers (Abouelnaga et al. 2021; Bauer 1966). Using a nutrient agar medium, antibacterial activity undergoes in vitro evaluation for all compounds against *Staphylococcus aureus* and *Bacillus subtilis* (Gram-positive bacteria), *Escherichia coli*, and* Pseudomonas aeruginosa* (Gram negative bacteria). For Gram-positive and Gram-negative bacteria, conventional medications included ampicillin and gentamicin. The solvent (negative) control utilized was DMSO. In tests against bacterial strains, the chemicals were examined at a dose of 15 mg/mL.

### Method of testing

Twenty to twenty-five milliliters of the sterilized media were added to each sterilized Petri dish, and they were left to harden at room temperature. The McFarland 0.5 standard solution was used to prepare the microbial suspension (1.5 × 105 CFU mL^−1^) with a turbidity of OD = 0.13 adjusted at a wavelength of 625 nm in a spectrophotometer. The inoculum suspension's turbidity was adjusted within 15 min, and then a sterile cotton swab was saturated on the dried agar surface and left for 15 min to dry with the lid in place. Using a sterile borer, 6-mm-diameter wells were created in the solidified material. Using a micropipette, 100 L of the tested compound's solution was added to each well. In order to test for antibacterial activity, the plates were incubated for 24 h at 37 °C. Inhibition zones were assessed on a millimeter scale throughout this experiment, which was done in triplicate. The MBC and MIC were established. MIC is defined as the lowest concentration of the antimicrobial agent that prevents the tested isolate from growing visibly when seen without the help of a microscope. The MIC broths used for MIC determination are subculture onto new agar plates to determine the MBC. MBC is the lowest drug concentration that successfully kills 99.9% of the tested microorganisms. The examined microorganism was acquired from the central laboratory of Cairo University’s faculty of science, Tanta University’s faculty of bacteriology, and Tanta University’s faculty of botany. The tested microorganisms were grown and maintained on nutrient agar. ATCC values of *Staphylococcus aureus* and *Bacillus subtilis* (Gram-positive bacteria), *Escherichia coli*, and* Pseudomonas aeruginosa* (Gram negative bacteria) are listed in Table 2.

**Table 2:** The type of strain of microorganisms

Name	Gram reaction	ATCC
*Bacillus subtilis*	G ^+^	6051
*Staphylococcus aureus*	G ^+^	12,600
*Escherichia coli*	G ^−^	11,775
*Pseudomonas aeruginosa*	G ^−^	10,145

### Molecular docking studies

#### Preparation

(a) Ligand preparation: This usually involves drawing or obtaining the 3D structure of the molecule (in this case, Schiff bases), followed by optimization using molecular mechanics algorithms. You would need to assign proper atomic charges, generate 3D conformations, and define rotatable bonds. (b) Protein preparation: Retrieve the 3D structure of the proteins (from databases like PDB). Remove any bound water molecules, add hydrogen atoms, assign correct charges, and determine the active site if not known. For our proteins of interest, the PDB IDs you mentioned (4k1p for *B. subtilis* and 7ab3 for *E. coli*) will be instrumental in this step.

#### Filtration

This usually means refining the list of ligands or conformations that will be used for docking. Not every ligand or conformation is viable or relevant. Filters might include removal of molecules with unfavorable ADME (absorption, distribution, metabolism, excretion) properties or toxicophoric.

#### Docking

(a) Choice of docking software: There are several docking tools available like Autodocking, Glide, DOCK, and GOLD. The choice depends on the complexity of the system and the accuracy required. (b) Grid generation: Define a grid around the protein active site. This grid will be used to explore different ligand positions and orientations*.* (c) Search algorithms: These help to predict the “best fit” conformation of the ligand in the protein active site. Algorithms can range from systematic searches to stochastic ones like genetic algorithms. (d) Scoring: Once a potential binding pose is found, a score is assigned based on the predicted binding affinity. The goal is to identify the orientation that maximizes the binding affinity (or minimizes the free energy of binding).

#### Analysis

(a) Binding pose visualization: Use molecular visualization software (like PyMOL or Chimera) to inspect the predicted binding orientation of the Schiff base within the protein. (b) Binding energy: Evaluate the binding affinity scores to assess how strongly the ligand may bind. (c) Interactions: Examine specific molecular interactions between the ligand and protein. This includes hydrogen bonds, van der Waals interactions, pi-pi stacking, and more. (d) Validation: Compare the docking results with experimental data if available. This could involve looking at known ligand binding orientations or experimentally determined binding affinities.

**Fig. 1:**
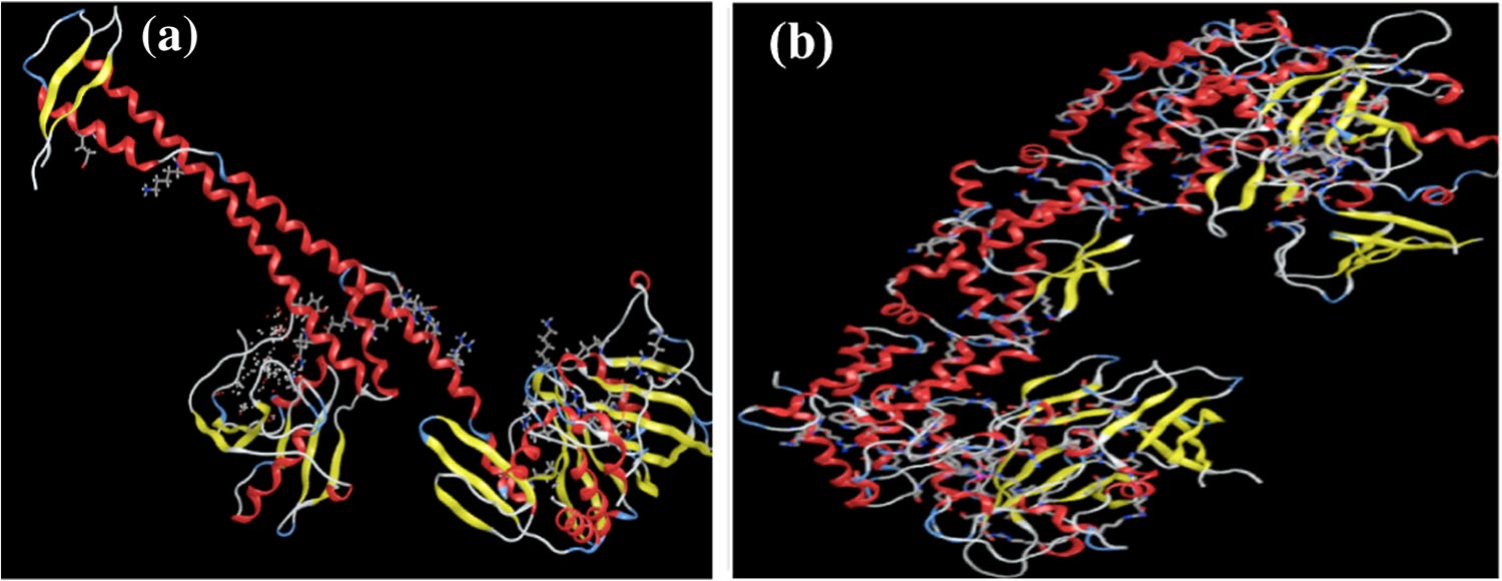
Amino acid residues side chain flexibility in **a** *Bacillus subtilis* (4k1p) and **b** *Escherichia coli* (7ab3) aureus proteins

*Bacillus subtilis* (4k1p) and* Escherichia coli* (7ab3) secondary structures were obtained from the Protein Data Bank of the Research Collaboratory for Structural Bioinformatics (RCSB; http://www.rcsb.org/) (Ghazy et al. 2023). The best of their possible ligand binding sites (cavity) were taken into consideration during geometry minimization technique (Table 3). Molecular docking’s maximum global minimization step was set at 1500 steps. These proteins’ side chain flexibility inside the cavity with tolerance of 1.00 and strength of 0.90 was taken into consideration (Fig. 1).

**Table 3:** Characteristics of the optimal cavity of the examined proteins for *Bacillus subtilis* (4k1p) and *Escherichia coli* (7ab3)

Protein	Radius	Center **	Volume	Surface area	Wt
*Bacillus subtilis* (4k1p)	15	X: 154.30, Y: 132.23, Z: 149.66	0.9 μm^3^	6 μm^2^	2.2 × 10^−13^ g
*Escherichia coli* (7ab3)	15	X: 0.00, Y: 4.35, Z: 0.00	0.6–0.7 μm^3^	8.0 μm^2^	

^∗∗^The MolDock grid score was set with a grid resolution of 0.30 Å

Afterwards, Molegro Virtual Docker (MVD) 6.01 was used to simulate molecular docking. The MolDock algorithm, a docking method based on guided differential evolution (combination of cavity prediction algorithm and differential evolution optimization methodology), identifies the proteins potential binding site and the direction of ligand binding for this purpose (Ghazy et al. 2023; Thomsen and Christensen 2006). Most of the time, docking precision is improved by reranking the top-ranked conformations (De Azevedo and Walter 2010). The MolDock scoring function iteration was set to 1500 with a simplex evolution size of 50 and a requirement of at least 10 runs. The simplex evolution also had 300 steps and a neighbor distance factor of 1.00.

### Dye removal

Methylene blue standard dye solutions were prepared and diluted with distilled water to the desired concentrations. To begin the adsorption procedure, the powder and nanofibers of the modified PMMA were introduced individually to 3 mL of the dye solution in a quartz cell at varied concentrations (0.1 and 0.05 g). The absorbance was recorded in the range of 190–1100 nm using UV/Vis spectrophotometer. At various times, the adsorption profile was observed. At the dye’s maximum wavelength, the absorption at any time (A_t_) and the absorption infinity (A_∞_) were measured. The initial absorbance (A_0_) was measured before the equilibrium of absorption, whereas (A_0_) was measured in the absence of solid adsorbing materials.

The final absorbance was measured, and using the equation, the percentage of dye elimination was computed.

$$\mathrm{\%Dye\;removal}=\left({\left[\mathrm{dye}\right]}_{\mathrm{t}}/{\left[\mathrm{dye}\right]}_{0}\times 100\right)$$

where [Dye]_t_ and [Dye]_0_ represent, respectively, the dye’s concentration at time *t* and its original concentration. The dye’s absorptivity was used to compute the concentration of the dye. Figures 13 and 14 reflect the concentration profiles for the removal of methylene blue dye using modified PMMA powder and nanofibers, as shown by % dye removal.

## Results and discussion

### Characterization of polymeric materials

#### Static light scattering of PMMA

**Fig. 2:**
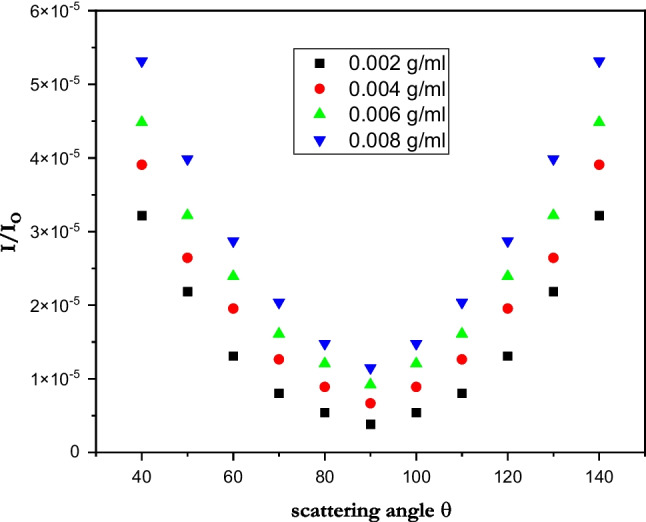
Angular distribution of the scattered light intensity

In addition to the second virial coefficient *A*_2_ and the radius of gyration *R*_G_, the PMMA molecular weight *M*_w_ was accurately determined using a static laser light scattering approach (Ghazy et al. 1999b, 2009). The calculation of polymer scattering parameters, which led to the measurement of the refractive indices of the various polymer concentrations (El-Baradie et al. 2000; Shaheen et al. 2018), heavily relies on the refractive index increment. In DMF, PMMA was dissolved at concentrations of 2, 4, 6, and 8 × 10^3^ g/ml, and the results of static light scattering tests were then analyzed. Secondly, a digital Abbe refractometer with a 0.0001 precision was used to test the refractive index of the various PMMA concentrations. The measured values of the refractive index were then used to calculate the refractive index increment as ${\left.dn/dc\right|}_{\mathrm{c}\to 0}=\underset{\mathrm{c}\to \mathrm{o}}{\mathrm{Lim}}\left(n-{n}_{\mathrm{o}}/c\right)$, where *n*, *n*_o_, and *c* are the solution refractive index, the solvent refractive index, and the concentration, respectively (Ghazy 2008; Ghazy et al. 1999a). The refractive index increment was found to be 0.067 ml/g. When measuring the intensity of the scattered light, an Oriel model 77344 photomultiplier tube was used to measure the angular distribution of the intensity of the scattered light for the prepared concentrations in the angle ranges of 40 to 140° (Fig. 2). The incident light source was a Nd-YAG laser with a wavelength of 650 nm.

**Fig. 3:**
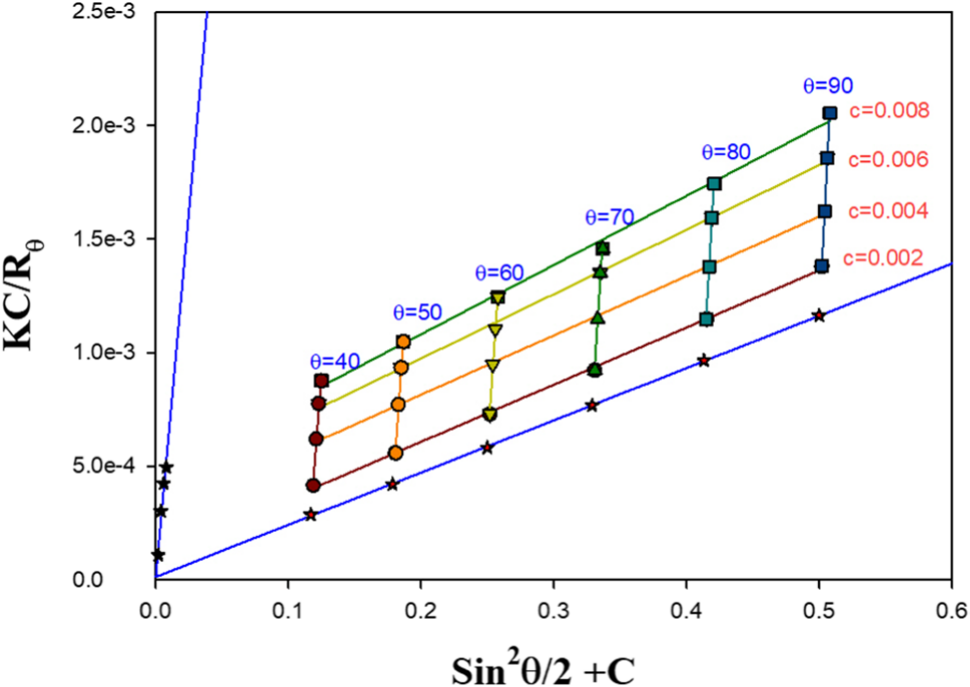
Zimm plot of PMMA

Zimm plot (Fig. 3) was constructed using the fundamental static light scattering equation to calculate the scattering parameters of molecular weight M_W_, second virial coefficient *A*_2_, and radius of gyration *R*_G_ as:

$$\frac{Kc}{{R}_{\theta }}=\frac{1}{{M}_{w}}\left[1+\left(\frac{16{\uppi }^{2}}{3{\uplambda }^{2}}\right){R}_{G}^{2}{\mathrm{sin}}^{2}\left(\frac{\uptheta }{2}\right)\right]+2{A}_{2}c$$

where $K=2{\uppi }^{2}{n}_{o}^{2}/{\uplambda }^{4}{N}_{\mathrm{A}}{\left(dn/dc\right)}^{2}\left(1+{\mathrm{cos}}^{2}\uptheta \right)$ and ${R}_{\uptheta }={I}_{\uptheta }{r}^{2}/{I}_{o}V$, where *λ* is the wavelength of the scattered light, ${N}_{A}$ is Avogadro’s number, θ is the scattering angle, ${n}_{o}$ is the solvent refractive index, ${I}_{\theta }$ and ${I}_{o}$ are the scattered and incident light intensities respectively, *r* is the distance between the scattering point and detector, and *V* is the scattering volume (Ghazy 2011; Shaheen et al. 2020). The calculated scattering parameters and refractive index increment value are tabulated in Table 4.

**Table 4:** Molecular weight of PMMA

	dn/dc (ml/g)	Mw (g/mol)	R_G_ (μm)	A_2_ (mol.cm^3^/g^2^)
PMMA	0.067	82,508	1.23	0.032

#### Fourier Transform Infra-Red Spectrum

**Fig. 4:**
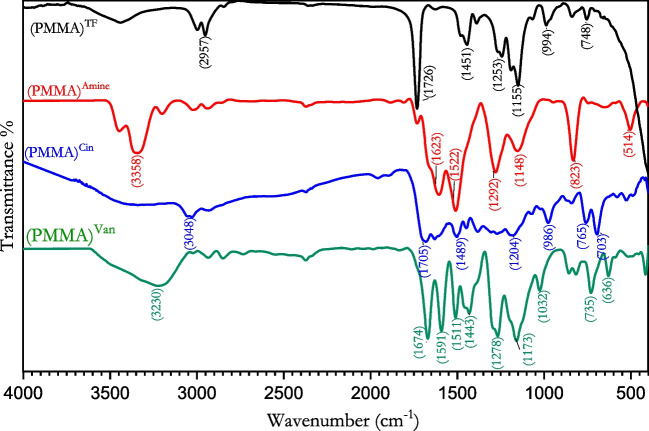
FT-IR of (PMMA)^TF^, (PMMA)^Amine^, (PMMA)^Cin^, and (PMMA)^Van^

PMMA's FT-IR spectra (Fig. 4) were notable for having a strong peak at 1119 cm^−1^ caused by O-C-O CH_3_’s stretching. The production of a (C = O) bond, which is responsible for the existence of a high peak at 1604 cm^−1^, shows the formation of a carbonyl group in PMMA (El-Aassar et al. 2019a; Haris et al. 2010). Due to CH_2_ stretching, the peak became visible at about 2950 cm^−1^ (Abdel-Aziz et al. 2023; Hong et al. 2006). The reaction with P-phenylene diamine is confirmed by the FT-IR spectra of animated PMMA, which detect the amine stretching group at 3342 cm^−1^ as a winged peak (Elzain et al. 2019). The peak at 1550 cm^−1^ further demonstrated the (C = N) group’s presence in the aminated PMMA. The following peaks are produced by the reaction of aminated PMMA with vanillin; peak at 1456 cm^−1^ corresponding to the C = C stretches in the aromatic ring in vanillin and cinnamaldehyde in the final copolymer, and peaks at 729 cm^−1^ and 1025 cm^−1^ corresponding to the in-plane C-H bending (El-Aassar et al. 2019b; Lee and Jang 1996). The C = N stretching accounts for the peak at 1670 cm^−1^. The presence of a large signal at 3225 cm^−1^ demonstrates that vanillin contains a hydroxyl group. This demonstrates that the Schiff base was indeed formed.

#### Thermogravimetric analysis

**Fig. 5:**
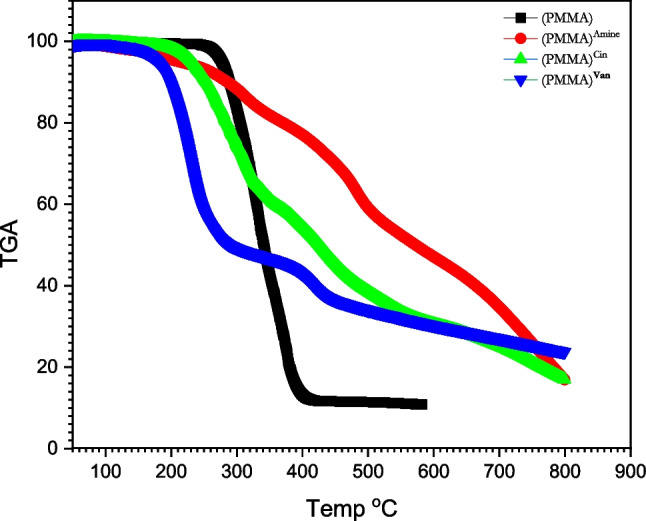
TGA of (PMMA), (PMMA)^Amine^, (PMMA)^Cin^, and (PMMA)^Van^

By observing the weight loss of the sample as a function of temperature, this analytical approach is used to assess the thermal stability of materials and the fraction of volatile components (Ghazy et al. 2022; Prime et al. 2009; Saadatkhah et al. 2020). Under a nitrogen environment, the samples were monitored at a steady rate of 20 °C/min between 30 and 800 °C. As the temperature rises, the sample loses weight faster. The sample displayed three separate stages of weight reduction, according to the TGA curves. (PMMA)^Van^ residual is 23.67 at 800 °C, 75% weight lost at 763.18 °C, 50% weight lost at 277.54 °C, and 25% weight lost at 212.75 °C, according to Fig. 5 and Table 5. It is therefore evident that (PMMA)^Van^ is more thermally stable than PMMA, which loses 25% of its weight at 364.41 °C and leaves no residue at 800 °C. At 800 °C, the (PMMA)^Cin^ residual is 16.96, the weight is reduced by 75% at 701.2 °C, 50% at 417.02 °C, and 25% at 287.6 °C. Hence, it is evident that (PMMA)^Cin^ is more thermally stable than PMMA because there is no leftover material at 800 °C and the weight loss at 75% is 364.41.

**Table 5:** Weight lost% for (PMMA), (PMMA)^Amine^, (PMMA)^Cin^, and (PMMA)^Van^

Weight lost %	25%	50%	75%	Residual at 800°C
PMMA	300.35	329.79	364.41	0
PMMA amine	416.5	572	755	16.88
PMMA Cin	287.6	417.02	701.2	16.96
PMMA Van	212.75	277.54	763.18	23.67

Moreover, (PMMA) ^Van^ is the most thermally stable material among (PMMA)^TF^, (PMMA)^Amine^, (PMMA)^Cin^, and (PMMA)^Van^, as shown in Fig. 5 and Table 5.

#### X-ray diffraction analysis

**Fig. 6:**
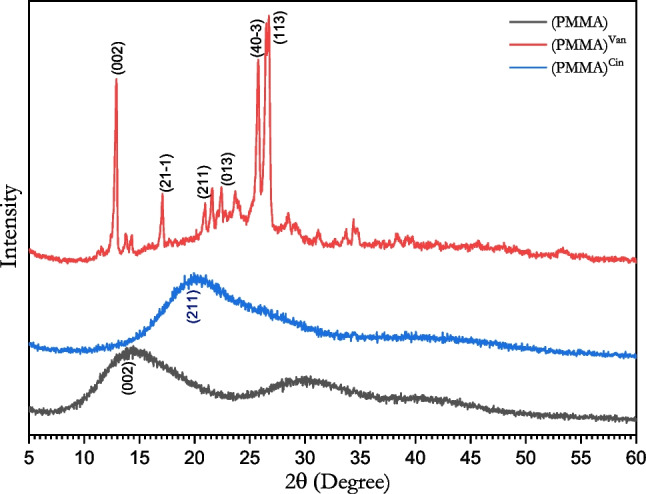
XRD of (PMMA), (PMMA)^Van^ and (PMMA)^Cin^

Figure 6 displays the XRD patterns of PMMA and its variations. As can be observed, PMMA exhibits three large peaks at 2 θ = 14.30°, 29.60°, and 40.50°, indicating that the material has an amorphous structure (Thakur et al. 2014). Although the structure is changed as a result of the reaction with cinnamaldehyde, which is shown by just one broad peak at 2 θ = 19.7°, the structure is still amorphous. In contrast, the crystalline structure was altered by the reaction with vanillin, as seen by the strong peaks at 2θ = 12.78°, 16.96°, 21.46°, 22.30°, 25.64°, and 26.56°. The crystalline size of (PMMA)^van^ was then calculated using the Debye–Scherrer equation (Holzwarth and Gibson 2011; Kalishwaralal et al. 2008), ${D}_{Av}=0.94\lambda /\beta \mathrm{cos}\theta$, where *λ* = 0.154 nm and *β* is in radians. Table 6 lists the measured diffraction peaks along with the matching crystallographic size, full width at half maximum FWHM (β), and interplanar spacing (d).

**Table 6:** X-ray diffraction analysis

Sample	Observed			Debye–Scherrer	
	2θ	*d* (Ǻ)	Hkl	*β*	*D*_Av_
Van PMMA	12.78	6.9239	002	0.2529	188.13
Monoclinic					
*a* = 13.70Ǻ	16.96	5.2244		0.3538	134.47
*b* = 7.50Ǻ	21.46	4.1366	211	0.2841	167.47
*c* = 15.50Ǻ	22.30	3.9842	013	0.7725	61.59
*α* = 90°					
*β* = 120°	25.64	3.4716		0.3464	13.73
*γ* = 90°					
*V* = 1380Ǻ^3^	26.56	3.3536	113	0.4955	9.600
Average					**95.83**

### Scanning electron microscopy

**Fig. 7:**
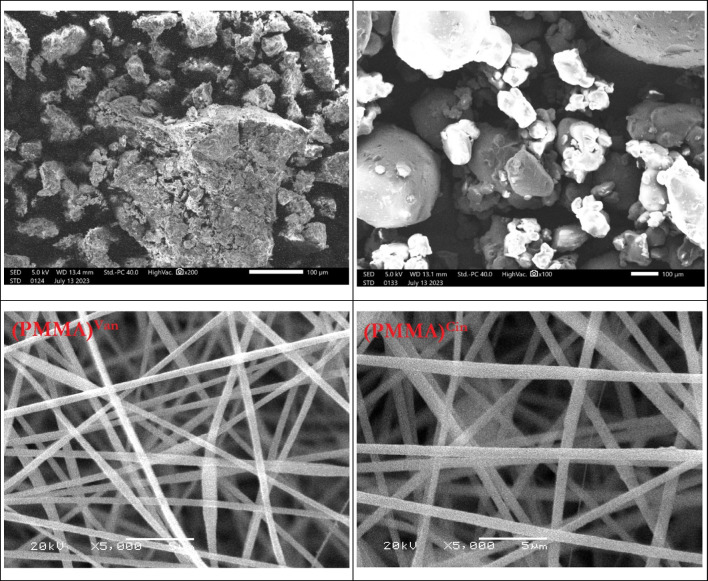
SEM images for (PMMA)^Van^ and (PMMA)^Cin^ powder and nanofibers

The final reaction products were fabricated in the form of nanofibers. Scanning electron microscope was used to demonstrate their influence on the morphological structure of the electrospun nanofibers compared to its powder origin (Fig. 7). SEM images of the (PMMA)^Van^ powder showed a total agglomeration of PMMA particles with vanillin particles. On the other hand, (PMMA)^Cin^ powder appeared as a blend between PMMA and cinnamaldehyde particles. The PMMA nanofibers diameter was found to be in the range of 500 nm. The morphological structure of the two compounds is typically similar except for the nanofiber diameter as the modification nanofiber size of the vanillin was 600 nm, whereas it was about 800 nm for the cinnamaldehyde.

### Antibacterial activity

A modified Kirby-Bauer disc diffusion method was used to assess the antibacterial activity of the tested materials (Bauer 1966; Midolo et al. 1995) (NCCLS (Wayne 2002), NCCLS (Chang et al. 1997)). Gram ( +) bacteria like *Staphylococcus aureus* and* Bacillus subtilis*, as well as Gram ( −) bacteria like *Escherichia coli* and* Pseudomonas aeruginosa*, were investigated, and it was discovered that the modified polymers and their nanofibers exhibited antibacterial activity.

#### Inhibition on linear growth of selected pathogenic bacteria

**Fig. 8:**
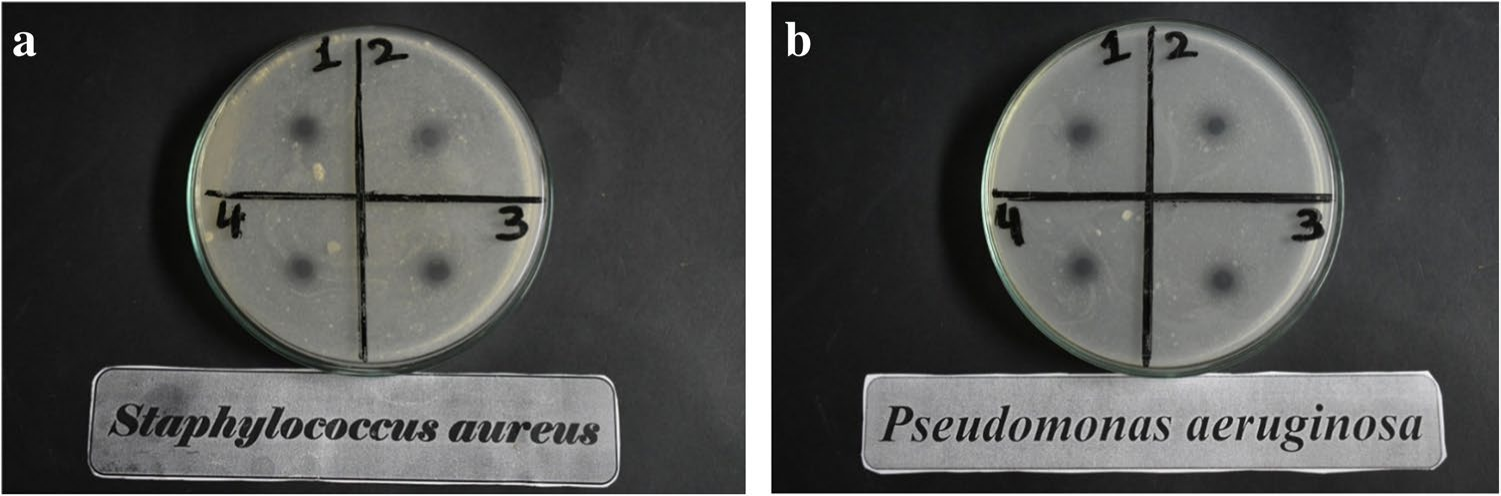
**a** Examples of the inhibition zones of linear growth of Gram-positive bacteria against modified PMMA (1) (PMMA)^Van^ powder, (2) (PMMA)^Van^ nanofiber, (3) (PMMA)^Cin^ powder, and (4) (PMMA)^Cin^ nanofiber. **b** Examples of the inhibition zones of linear growth of Gram-negative bacteria against modified PMMA (1) (PMMA)^Van^ powder, (2) (PMMA)^Van^ nanofiber, (3) (PMMA)^Cin^ powder, and (4) (PMMA)^Cin^ nanofiber

Figure 8 and Table 7 display the antibacterial activity of modified PMMA powder when used (0.8 g) against several types of harmful bacteria with gram ( +) and gram ( −) ratings. With respect to Gram-positive bacteria (*B. subtilis* and* S. aureus*), (PMMA)^Van^ gave 10, 12, and 26, 21 mm, respectively, when compared to ampicillin as an antibacterial standard. *E. coli* and* P. aeruginosa*, which are Gram-negative bacteria, gave results of 12 and 12 mm, respectively. (PMMA)^Cin^ yielded (9, 10) and (11, 10) mm, respectively, when tested against G ( +) and G ( −) bacteria.

**Table 7:** Diameters of inhibition zones (mm) by using 0.8 g of modified PMMA powder

Sample	Inhibition zone diameter (mm/mg Sample)			
	G^+^		G^−^	
	*PA*	*EC*	*SA*	*BS*
Control: DMF	0.0	0.0	0.0	0.0
Standard: (ampicillin) antibacterial agent	26	25	21	26
(PMMA)^Van^	12	12	12	10
(PMMA)^Cin^	10	11	10	9

*BS Bacillus subtilis*, *SA Staphylococcus aureus*, *EC Escherichia coli*, and* PA Pseudomonas aeruginosa*

**Fig. 9:**
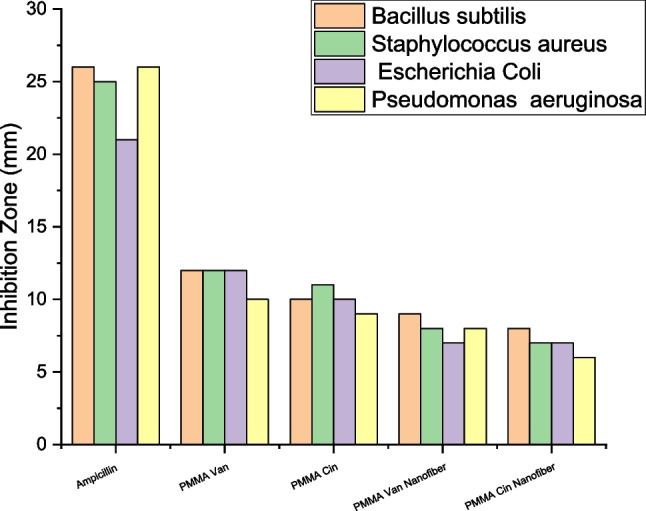
Inhibition zone on linear growth of modified PMMA against the selected pathogenic bacteria

Using 0.2 g of modified PMMA nanofiber versus various kinds of Gram ( +) and Gram ( −) harmful bacteria, Table 8 demonstrated the antibacterial activity of the material. In comparison to ampicillin as an antibacterial standard, (PMMA)^Van^ nanofiber gave 8 and 7 mm, respectively, against Gram positive bacteria (*Bacillus subtilis* and* S. aureus*). While *E. coli* and* P. aeruginosa*, two Gram-negative bacteria, produced (8 and 9 mm, respectively). (PMMA)^Cin^ nanofibers exhibited (6 and 7) and (7 and 8 mm) results against G ( +) and G ( −) bacteria, respectively. The values of the inhibition zones found to be compatible with Preda et al. which studied the antimicrobial of PMMA-doped ZnO NPs and the inhibition zone found to be ranged between 7 and 9 mm (Preda et al. 2023). Based on that finding, the antimicrobial activity of the nanofibers was found to be nearly the same of the modified PMMA powder despite using the quarter of the content in the nanofibers. The diameter of the inhibition zone for various compounds and bacteria are summarized in Fig. 9.

**Table 8:** Diameters of inhibition zones (mm) by using (0.2 g) of modified PMMA nanofibers

Sample	Inhibition zone diameter (mm/mg Sample)			
	G^+^		G^−^	
	*PA*	*EC*	*SA*	*BS*
Control: DMF	0.0	0.0	0.0	0.0
Standard: (ampicillin) antibacterial agent	26	25	21	26
(PMMA)^Van^	9	8	7	8
(PMMA)^Cin^	8	7	7	6

*BS Bacillus subtilis*, *SA Staphylococcus aureus*, *EC Escherichia coli* and* PA Pseudomonas aeruginosa. G* Gram reaction, *Solvent* DMF

### Determination of MIC and MBC

**Fig. 10:**
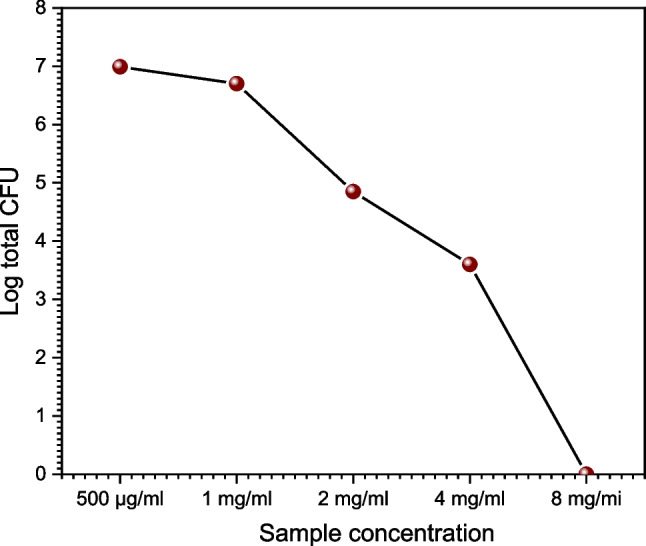
Log total CFU

As shown in Table 9 and Fig. 10, increasing the concentration of the modified PMMA (500/ml, 1, 2, 4, 8 mg/ml) increases the inhibitory effect. While the Log total CFU was 6.70 at a concentration of 1 mg/mL and 6.99 at a concentration of 500/ml, it was only 4.85 at a concentration of 2 mg/mL. *Streptococcus mutans* (G-) was used as the control, and the percentage was 3.6 with concentrations of 4 mg/mL and 8 mg/mL, respectively. The outcome showed that the minimum inhibitory concentration (MIC) and minimum bactericidal concentration (MBC) quantitatively determined the growth inhibition effect (MBC). According to the findings, the inhibitory effect grows as the modified PMMA concentration does.

**Table 9:** Determination of MIC and MBC

Streptococcus mutants	8 mg/ml (MBC)	4 mg/ml	2 mg/ml (MIC)	1 mg/ml	500μg/ml
Volume of broth plated (μl)	100 μl	100 μl	100 μl	20 μl	20 μl
Colony forming unit (CFU)/plate	0	2	35	500	980
Total CFU /ml	0	4000	70,000	5 × 106	9.8 × 106
Log total CFU	0	3.60	4.85	6.70	6.99

The stock concentration of PMMA Van drug 16 mg/mL

The minimum inhibitory concentration (MIC) of the (PMMA)^Van^ sample is 2mg/mL

The minimum bactericidal concentration (MBC) of the (PMMA)^Van^ sample is 8 mg/mL

### Docking calculation

**Fig. 11:**
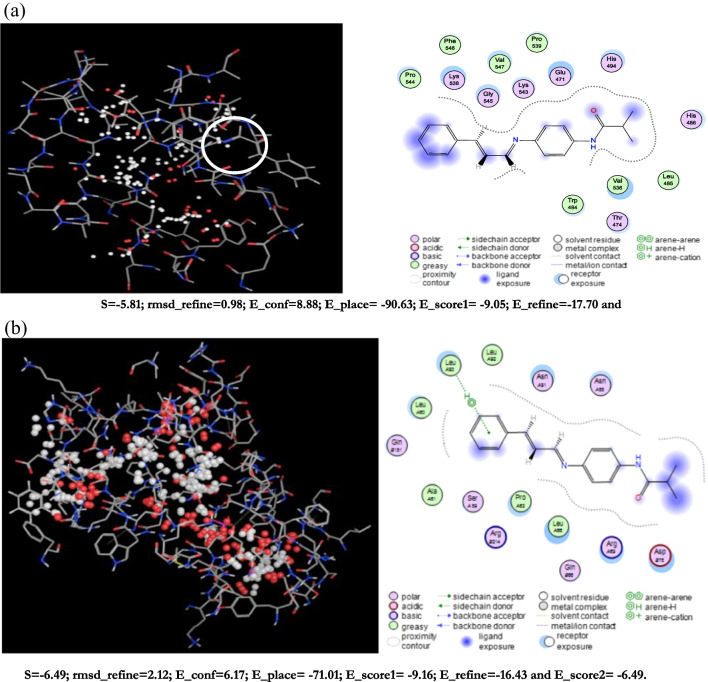
**a** The potential ligand binding cavity, molecular interaction between ligand and active site of *Bacillus subtilis* (4k1p) with flexible residues of (PMMA)^Van^. **b** The potential ligand binding cavity, molecular interaction between ligand and active site of *Escherichia coli* (7ab3) with flexible residues of (PMMA)^Van^

**Fig. 12:**
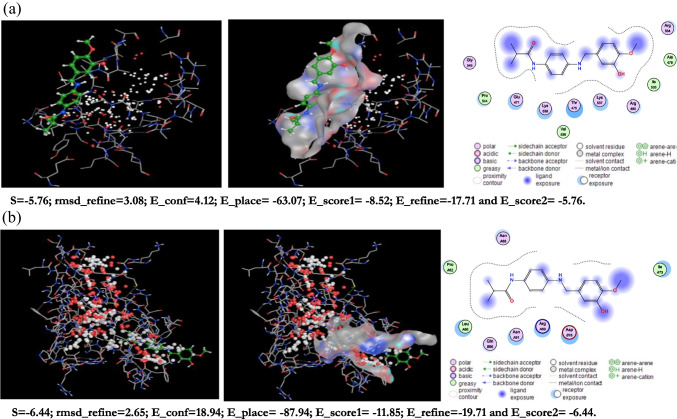
**a** The potential ligand binding cavity, molecular interaction between ligand and active site of *Bacillus subtilis* with flexible residues of (PMMA)^Cin^. **b** The potential ligand binding cavity, molecular interaction between ligand, and active site of *Escherichia coli* (7ab3) with flexible residues of (PMMA)^Cin^

The ideal binding cavity for each molecular docking simulation utilizing the MVD tool suite is shown in Figs. 11 and 12. Table 10 displays the docking scoring results for the best cavity. A more negative re-rank score (7ab3) denotes more ligand docking into the active site of *Escherichia coli* (7ab3) and *Bacillus subtilis* (4k1p), respectively. This table shows that *Bacillus subtilis* protein (4k1p) is larger than *E. coli* protein, giving *S. aureus* a higher negative re-rank score than *E. coli* (7ab3). The molecular interaction energy between the proteins (4k1p), (7ab3), and the ligand is 92.05 and 87.25 kJ mol^1^, respectively. Additionally, the (4k1p) and (7ab3) aureus proteins’ binding pockets contain deep bonded and non-bonded interaction ligands, which point to a strong molecular connection between the two contacts (Figs. 11a and 12a). 4k1p and (7ab3) interact with Arg33, Lys36, Ala40, and Arg41 despite these interactions being with Glu38, Ala40, His57, and Gly56. These interactions at the ligand’s active site are shown in Figs. 11b and 12b.

**Table 10:** Molecular docking score of *Bacillus subtilis* (4k1p) and *Escherichia coli* (7ab3) proteins with ligand in the favorable cavity

Protein–ligand	RS	*E*_tot_	IE	*E*_HB_
*Escherichia coli* (7ab3) + (PMMA)^Van^	− 5.81	− 8.88	− 17.70	− 5.82
*Bacillus subtilis* (4k1p) (PMMA)^Van^	− 6.49	− 6.17	− 16.43	− 6.49
*Escherichia coli* (7ab3) + (PMMA)^Cin^	− 5.76	− 4.12	− 17.71	− 5.76
*Bacillus subtilis* (4k1p) (PMMA)^Cin^	− 6.44	− 18.94	− 19.71	− 6.44

*RS* Re-rank score is linear combination of internal (Steric, van der Waals, hydrogen bonding, and electrostatic) and external (torsion strain, torsion strain sp2-sp2, hydrogen bonding, van der Waals, and electrostatic) energies, *Etot* total interaction energy between protein and pose, *IE* internal energy of pose, *EHB* hydrogen bonding energy

In fact, proteins with additional active sites bind ligand molecules in the binding region rather firmly. This result is in line with the properties of chemical reactivity. On the energy maps of *Bacillus subtilis* (4k1p) and* Escherichia coli* (7ab3) proteins, green, turquoise, yellow, red, and blue may be responsible for favorable steric interactions, hydrogen acceptor, hydrogen donor, and electrostatic potential with the ligand.

**Fig. 13:**
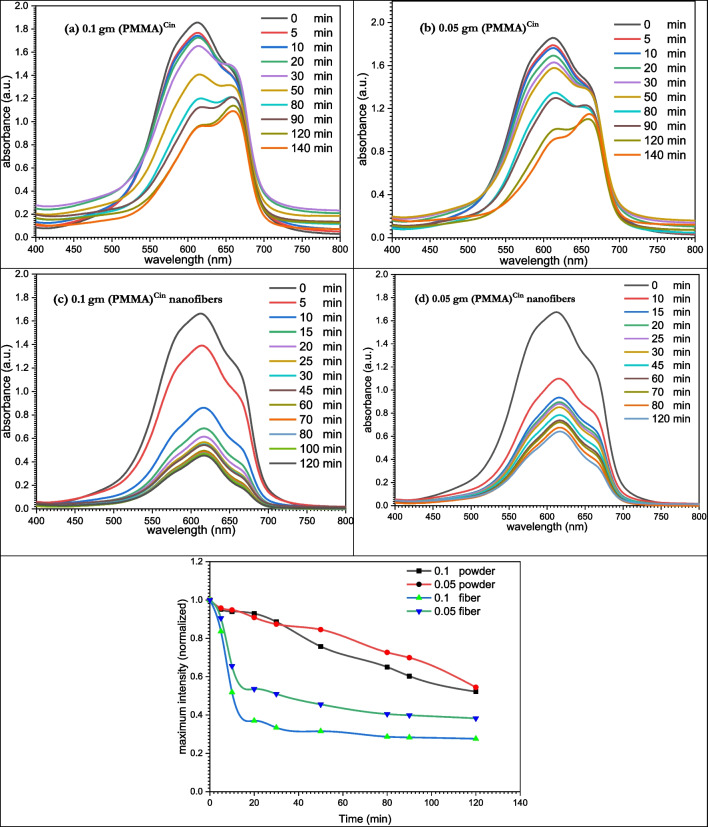
Dye removal curves using (PMMA)^Cin^

**Fig. 14:**
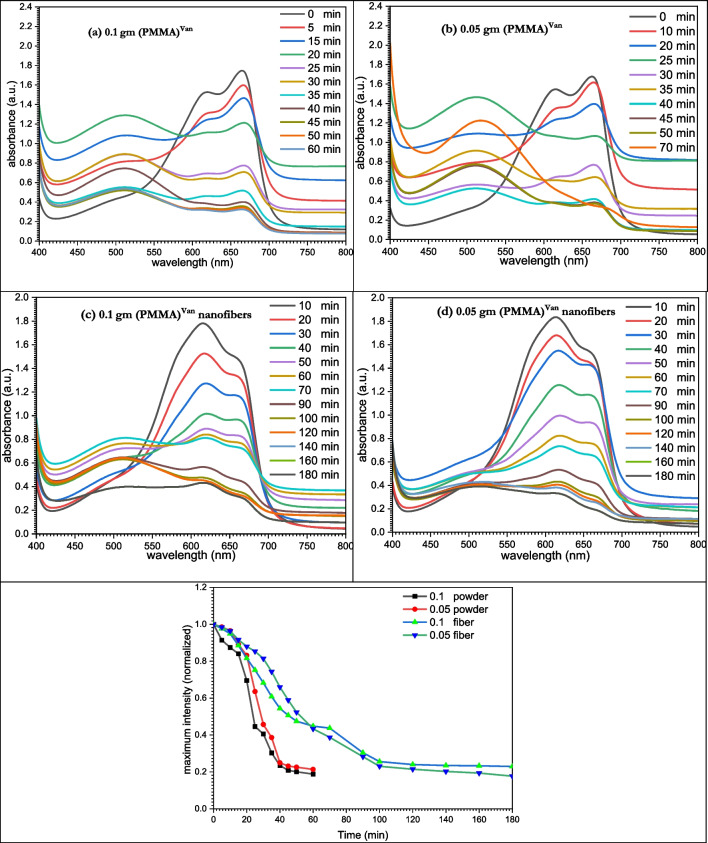
Dye removal curves using (PMMA)^Van^

In contrast to steric, hydrogen donor, and electrostatic potential interactions, electrostatic potential interaction is the most probable in *Escherichia coli* (7ab3) (Figs. 13b and 14b). According to our research, the binding cavities of proteins from *Escherichia coli* (7ab3) and* Bacillus subtilis* (4k1p) have a favorable ligand–protein interaction energy. Moreover, one may contend that this ligand functions as an efficient inhibitor (De Azevedo and Walter 2010).

### Dye removal

Due to the potentially harmful consequences on the environment, the adsorption of dyes by PMMA and its modified materials has been the focus of numerous study (Cantarella et al. 2016; Rizzo et al. 2007; Wen et al. 2022). For the purpose of eliminating methylene blue (MB) from an aqueous solution, adsorption tests were conducted. The absorbance at the highest wavelength is displayed against time in Figs. 13 and 14, which demonstrate spectrophotometric monitoring of the adsorption processes.

#### Using (PMMA)^Cin^

When methylene blue was removed using 0.1 g (PMMA)^Cin^, the absorbance of the dye fell from 1.5 a.u. to 1.3 a.u. after 30 min but remained constant at 1.06 a.u. after 2 h (Fig. 13a). As the amount of (PMMA)^Cin^ was reduced to 0.05 g, the target dye’s absorbance increased to 1.41 a.u. after 30 min and stabilized at 1.1 a.u. after 2 h (Fig. 13b).

The absorbance of the dye decreased from 1.29 a.u. to 0.34 a.u. after 30 min of methylene blue removal using 0.1 g (PMMA)^Cin^ nanofibers but remained constant at 0.25 a.u. after 2 h (Fig. 13c). The target dye’s absorbance climbed to 0.64 a.u. after 30 min and stabilized at 0.42 a.u. after 2 h as the amount of (PMMA)^Cin^ nanofibers was decreased to 0.05 g (Fig. 13d).

When (PMMA)^Cin^ powder was used, 26.67% of the MB dye was removed; however, the percentage of dye removal was 80.62% when (PMMA)^Cin^ nanofibers were employed.

#### Using (PMMA)^Van^

After removing methylene blue for 30 min with 0.1 g (PMMA)^Van^, the dye’s absorbance dropped from 1.62 a.u. to 0.66 a.u. but stayed constant at 0.31 a.u. after 70 min (Fig. 14a). While the dose of (PMMA)^Van^ was reduced to 0.05 g, the target dye’s absorbance increased to 0.76 a.u. after 30 min and stabilized at 0.36 a.u. after 70 min (Fig. 14b).

When employing 0.1 g (PMMA)^Van^ nanofiber, the methylene blue dye’s absorbance was lowered from 1.6 a.u. to 0.48 nm after 90 min, and it stayed constant at 0.34 a.u. after 3 h (Fig. 14c). The target dye’s absorbance increased to 0.45 a.u. after 90 min when the amount of (PMMA)^Van^ nanofiber was decreased to 0.05 g, and it stabilized at 0.23 a.u. after 3 h (Fig. 14d). With 0.05 g (PMMA)^Van^ powder and 0.05 g (PMMA)^Van^ nanofibers, the clearance rate of MB dye was 78.44% and 85.63%, respectively.

## Conclusion

Precipitation polymerization was used to produce poly methyl methacrylate (PMMA), which was subsequently aminated and reacted with the Schiff bases vanillin and cinnamaldehyde. Static light scattering analysis of the synthesized polymer reveals a molecular weight of 82,508 g/mol. PMMA, (PMMA)^Van^, and (PMMA)^Cin^ were characterized using FT-IR, TGA, XRD, and SEM techniques. (PMMA)^Van^ is the most thermally stable material. PMMA and (PMMA)^cin^ have amorphous structures, whereas (PMMA)^van^ has crystalline structures. Despite using only 25% of the material in the fiber, PMMA nanofiber was found to have almost the same antibacterial activity as PMMA powder. The binding cavities of proteins from *Escherichia coli* (7ab3) and* Bacillus subtilis* (4k1p) have a favorable ligand–protein interaction energy, according to our findings. When (PMMA)^Cin^ powder was utilized, 26.67% of the MB dye was removed; however, when (PMMA)^Cin^ nanofibers were used, the percentage of dye removal was 80.62%. The clearance rate of MB dye was 78.44% with 0.05 g (PMMA)^Van^ powder and 85.63% with 0.05 g (PMMA)_Van_ nanofibers, respectively. According to the results, modified PMMA powders and nanofibers can be employed as antibacterial agents and dye removers in water treatment applications.

## Data Availability

No data was used for the research described in the article. The data presented in this study are available in the article.
